# Are Dents in the Forehead Inherited?

**Published:** 1916-03

**Authors:** Leonard Keene Hirshberg

**Affiliations:** Johns Hopkins


### Are Dents in the Forehead Inherited?

BY DR. LEONARD KEENE HIRSHBERG, A. B., M. A., M. D. (JOHNS HOPKINS).

So at least thinks Professor Charles B. Davenport. He says one of the most striking phenomena to a student of heredity is the definiteness with which certain small, apparently insignificant, peculiarities are inherited. This is very prettily illustrated again by a case which a correspondent has sent to Dr. Davenport, and, although the family history is only a fragment, it is so instructive as to be worth mentioning.

The trait in question consists of a small depression in the sagittal plane of the frontal bone, extending two or three centimeters above a line joining the upper limits of the orbits. The depression is a striking one, but the morphological changes involved do not seem to be great. Nevertheless they are very persistent in the family history and without doubt indicate a definite modification of the germ plasm which is, in accordance with the modern interpretation of the positive, dominant sort; that is, due to the addition of a gene.

Starting with a fraternity of three grown children, born between 1885 and 1890, the first, a male, has a deep dent in his forehead, as if his skull were pushed in. The second, male, has a deep crease or dent in forehead, and the third, female, has a crease in forehead. A fourth member of the fraternity was a small boy, who died at the age of nine months, and of whom no description was given. The *mother* was unrelated to the father and none of her relatives had a peculiarity of this sort. By another wife the father had one daughter, who, likewise, had the crease in the forehead. As this other wife was unrelated and did not show the crease in the forehead, this portion of the pedigree, alone, is sufficient to suggest strongly that the positive trait is carried in the germ plasm of the father.

The father was one of a fraternity of seven, three males and four females. One of his brothers showed the same crease in the forehead. The other did not. Three of the sisters certainly have shown the crease in the forehead. One of these is married and had six children, one boy and five girls, and of these girls three show the crease in the forehead. Of the fourth sister of the father, long since dead, there are no precise data. In response to an inquiry, a group of photographs were examined in which the sister appears and said that it is impossible to be sure, but there was a slight trace of it from what she could see. This fourth sister had four children (by three husbands), and one (a son) shows the crease in the forehead. The single member of the second generation who lacks the family trait has two children, neither of whom has the trait; however, the same is true of the two children of his sister, who has the trait. Unfortunately, definite knowledge is extant concerning two generations only. The conditions in the grandparents are unknown. So far, then, as the information goes it indicates that this slight family peculiarity in the form of the frontal bone of the skull is inherited as a dominant trait.

In conclusion he wishes to request those in whose family such (or other clearly marked) peculiarity in the form of the skull appears, or who know of such a family trait among their acquaintances, to communicate the fact to him.

---

Texas travelers who use Dallas' new union station will be assured of first-class medical service if they are taken ill while within its precincts. The station is to have an emergency hospital for first-aid treatment. It will occupy quarters on the ground floor at the north end of the station near the drug store. All the latest surgical devices will be installed and a trained attendant will be on hand.

---

The "Simpson light," named after its discoverer, is based on the affinity of rare metals for each other. Two kinds of rays are produced—visible and invisible. The light is said to have produced marked benefits in the treatment of diseases of the nose and throat as well as in the case of skin diseases. The vapor from the lamp also helps asthma. But it will take some time to see whether these cures are permanent.

---

Under the present city administration and board of health, Parkland Hospital, which is the only charity hospital in Dallas, has undergone many important changes. The poor receive there the best care and attention that is possible to give the sick. A Mexican ward and a negro ward have been added, a children's ward opened and a nurses' training school established. And the staff of physicians include the best doctors in the city. The woman's committee is endeavoring to found a library for the hospital.

